# Repetitive Mild but Not Single Moderate Brain Trauma Is Associated with TAR DNA-Binding Protein 43 Mislocalization and Glial Activation in the Mouse Spinal Cord

**DOI:** 10.3390/biomedicines13010218

**Published:** 2025-01-16

**Authors:** Tamara Janković, Jelena Rajič Bumber, Nika Gržeta Krpan, Petra Dolenec, Marc Jaeger, Jasna Kriz, Gordana Župan, Kristina Pilipović

**Affiliations:** 1 Department of Basic and Clinical Pharmacology and Toxicology, Faculty of Medicine, University of Rijeka, 51000 Rijeka, Croatia; tamara.jankovic@medri.uniri.hr (T.J.); jelena.rajic@medri.uniri.hr (J.R.B.); nika.grzeta.krpan@medri.uniri.hr (N.G.K.); petra.dolenec@medri.uniri.hr (P.D.); 2 Department Chirurgie, Spital Oberengadin, CH-7503 Samedan, Switzerland; marc@jaeger-hh.de; 3 Department of Psychiatry and Neuroscience, Faculty of Medicine, Université Laval, Quebec City, QC G1V 0A6, Canada; jasna.kriz@fmed.ulaval.ca; 4 Independent Researcher, 51000 Rijeka, Croatia; gordana.zupan@gmail.com

**Keywords:** traumatic brain injuries, cord, spinal, TDP-43 proteinopathies, microglial cells, neuroinflammation

## Abstract

**Background/Objectives**: Traumatic brain injury (TBI) occurs after a sudden mechanical force to the skull and represents a significant public health problem. Initial brain trauma triggers secondary pathophysiological processes that induce structural and functional impairment of the central nervous system, even in the regions distant to the lesion site. Later in life, these changes can be manifested as neurodegenerative sequalae that commonly involve proteinopathies, such as transactive DNA-binding protein 43 (TDP-43). The progression of pathophysiological changes to the spinal cord motor neurons has been detected after repetitive TBI, while such changes have been less investigated after single TBI. **Methods**: Single TBI was applied over the left parietal cortex of mice by using the lateral fluid percussion injury apparatus and a separate cohort of animals received repetitive mild TBI by weight drop apparatus, with two mild injuries daily, for five days in a row. Mice were sacrificed after single moderate or last mild TBI and their spinal cords were prepared for the analyses. For both types of injury, sham-injured mice were used as a control group. **Results**: Here, we found an early formation of toxic phosphorylated TDP-43 species on the 3rdday post-injury which, together with TDP-43 cytoplasmic translocation, remained present in the subacute period of 14 days after repetitive mild but not single moderate TBI. During the subacute period following a repetitive brain trauma, we found an increased choline acetyltransferase protein expression and significant microgliosis in the cervical part of the spinal cord, which was not detected after single TBI. Astrogliosis presented similarly after both experimental procedures. **Conclusions**: This study demonstrates the differences in the spinal cord TDP-43 pathology and inflammation, depending on the brain trauma type, and may contribute to the development of targeted therapeutic strategies.

## 1. Introduction

Traumatic brain injury (TBI) is a condition caused by a sudden mechanical force to the skull. The primary impact triggers pathophysiological processes which develop over time, result in secondary pathology and are presented as structural and functional impairment of the central nervous system (CNS) [1,2]. TBI represents a significant and growing public health problem, especially in terms of population growth and ageing [3]. The Global Burden of Disease study pointed out that annually an estimated 55 million people experience TBI worldwide, with 8.1 million living with disability [3]. Depending on the injury severity, TBI survivors have lifelong consequences and require numerous neurorehabilitation procedures, from early rehabilitation to reintegration support [4].

Following brain trauma, a clinical examination determines the degree of the injury and severity of outcome using the Glasgow Coma Score (GCS) [5,6], whereupon TBI is subdivided into mild, moderate and severe. Although it often remains undiagnosed, mild head trauma is the most common type of TBI. In professional contact sports athletes [7,8,9], military personnel [10,11] and domestic violence victims [12], mild TBI is often repetitive. In terms of frequency, moderate and severe TBI follow and mainly occur as a result of traffic accidents and falls [5,13,14].

Studies have shown some differences in pathophysiological processes after single and repetitive TBI [15,16,17], but both injury types cause pathophysiological changes that can manifest as different neurodegenerative entities later in life [18,19,20,21,22]. TBI accelerates the development of all dementia types [22,23], but most frequently Alzheimer’s (AD) and Parkinson’s disease (PD) [16,22,24], amyotrophic lateral sclerosis (ALS) [25], frontotemporal dementia and chronic traumatic encephalopathy (CTE) [9,26]. Neurodegenerative sequelae of TBI commonly include proteinopathies, including TAR DNA-binding protein 43 (TDP-43) proteinopathy [15,27,28].

TDP-43 proteinopathy is considered a major pathological feature in CTE and ALS, while it appears as a secondary pathological feature in AD, PD and dementia with Lewy bodies [29,30]. TDP-43 is a ubiquitous protein, dominantly located in the nucleus, where it regulates almost 30% of the total transcriptome [31]. In the nucleus, TDP-43 regulates alternative splicing, mRNA life cycle and synthesis and processing of non-coding RNAs [30,32]. Normally, TDP-43 can shuttle between the nucleus and the cytoplasm to regulate nuclear transport and form ribonucleoprotein granules [21,33]. Conditions of cellular stress, such as oxidative stress and inflammation, promote cytoplasmic TDP-43 accumulation and post-translational modifications, such as cleavage and hyperphosphorylation, followed by the formation of aggregates [23,30,34]. In ALS patients, TDP-43 in neurons and glia of the cortex, but also brainstem and spinal cord, show permanent cytoplasmic translocation and formation of pathological aggregates, causing TDP-43 to be deficient in its physiological functions [35,36]. TDP-43 proteinopathy is commonly associated with repetitive head traumas [37], while there is some disagreement between experimental [38,39,40,41] and human studies [15,27,41] regarding its occurrence after a single brain trauma. TDP-43 inclusions have been dominantly investigated in different brain regions, but in cases of repetitive TBI, associated with ALS and CTE, TDP-43 aggregates were also detected in the spinal cord [28]. Single head injury has been related to the spinal cord motor neuron functionality impairment [38] but, to our knowledge, TDP-43 proteinopathy in the spinal cord after a single TBI has been investigated in only one experimental study [42].

The aim of this study was to clarify the incidence and extent of TDP-43 expression and its pathological forms in the cervical part of the spinal cord of mice exposed to a single moderate or repetitive mild TBI, in the early post-injury period, as well as in the subacute timepoint of 14 days after each type of brain trauma. In the aforementioned experimental groups, and the same spinal cord region, at the subacute timepoint, we also investigated glial activity and choline acetyltransferase (ChAT) expression, as well as TDP-43 changes in neurons and glia after an individual experimental TBI protocol.

Here, we report a significant pathophysiological difference in the response of the cervical part of the spinal cord between a single moderate and repetitive mild TBI. Acute phosphorylated TDP-43 formation, with prominent subacute cytoplasmic TDP-43 translocation and formation of toxic TDP-43 phosphorylated species, was detected after repetitive mild TBI, but not after a single moderate brain trauma. Also, an increased ChAT protein expression, in the cervical part of the spinal cord, was observed subacutely after repetitive mild, but not single moderate, TBI. Subacute microglial activation was detected only in the central canal of the cervical part of the spinal cord after a single moderate brain injury. Contrary to that, an extensive microglial reaction was observed in the central canal and the white matter regions after repetitive mild brain injuries, and was accompanied by an increase in the cytosolic IκBα expression. Following both experimental procedures, astrogliosis presented similarly, including white and grey matter regions, without changes in astrocytic TDP-43 localization.

## 2. Materials and Methods

### 2.1. Animals

The study utilized adult male wildtype C57BL/6 mice, sourced from the Laboratory of Mouse Engineering and Breeding Facility (LAMRI) at the University of Rijeka, Faculty of Medicine. Both maintenance and experimental procedures were conducted at the animal facility of the Department of Basic and Clinical Pharmacology and Toxicology at the same institution. The mice were housed under controlled temperature and humidity conditions, maintained on a 12 h light–dark cycle and provided with *ad libitum* access to water and standard rodent chow. The mice were randomly assigned to either control or trauma groups by blinded laboratory staff. To promote social interaction and reduce stress, mice were housed in groups of 3–5 per cage.

The experimental design adhered to the 3R principles (replace, reduce, refine) and complied with current institutional, national (NN 135/06, 37/13, 125/13, 55/13, 39/17) and international (European Parliament Directive 2010/63/EU) guidelines for the use of animals in experimental studies. All procedures were approved by the Faculty’s Ethical and Animal Welfare Committees and the Croatian Ministry of Agriculture on 7 January 2015 and 21 March 2017.

### 2.2. Experimental Traumatic Brain Injuries

In this study, two distinct models of experimental brain trauma were employed. The lateral fluid percussion injury (LFPI) method was utilized to induce a single moderate brain trauma, and the mice subjected to this type of injury will be referred to as the LFPI group. Conversely, the weight drop (WD) method was applied to induce repetitive mild traumatic brain injuries (rmTBI) in a separate cohort of mice, which will henceforth be designated as the rmTBI group.

A single moderate TBI was induced using LFPI apparatus adapted for adult mice according to the protocols described by Alder et al. [43] and Carbonell et al. [44]. Anesthesia was induced with isoflurane (3.5% for induction, 1.2–1.5% for maintenance), and the mice were secured in a stereotactic frame (David Kopf Instruments, Tujunga, CA, USA). Lubricant eye drops (Systane^®^ Gel Drops, Alcon, Geneva, Switzerland) were applied to protect the corneas. Normothermic body temperature was maintained using a heating lamp positioned above the mouse, and a blanket was used to keep the body warm during surgery. A craniotomy was performed over the left parietal cortex, 2.3 mm lateral to the midline and equidistant between the lambda and bregma sutures. A modified Luer Lock needle hub, with an internal diameter of 2.5 mm, was affixed over the intact dura mater using dental acrylic glue and cement. The LFPI device (VCU Biomedical Engineering Facility, Richmond, VA, USA) was connected to the Luer Lock via a 50 cm tube (ref. no. 8255172; B. Braun, Melsungen, Germany). Impact parameters were recorded using a pressure transducer and an oscilloscope, delivering a pressure pulse lasting 50 ms to induce moderate TBI. Post-injury, mice were briefly re-anesthetized for wound closure. Sham controls underwent identical procedures without head injury induction. All animals had uncompromised dura mater, adhering to exclusion criteria. Post-procedure, mice were returned to their home cages for recovery without observed respiratory complications or seizures.

**Figure 1:**
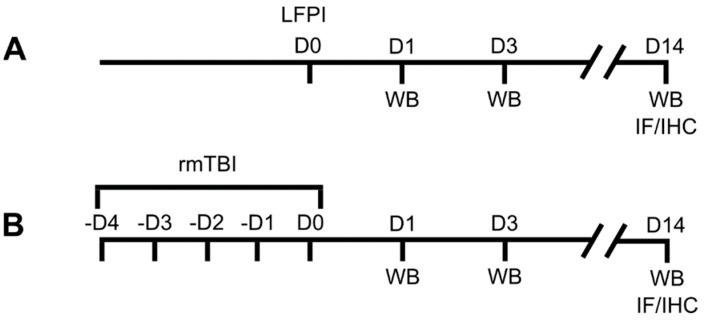
Experimental procedure timelines of mice subjected to (**A**) single moderate lateral fluid percussion injury (LFPI) or (**B**) repetitive mild traumatic brain injury (rmTBI). Animals belonging to the LFPI or rmTBI group were sacrificed 1, 3 or 14 days after the experimental protocol. Sham control mice of the LFPI or rmTBI group were euthanized on day 1 after the experimental protocol. Separate cohorts of mice were used for Western blotting (WB) and immunofluorescent/immunohistochemical analyses (IF/IHC). D: day.

Repetitive mTBIs were induced using the WD model, as described by Kane et al. [45] and previously utilized in our research. For each mild injury, the adult mice were lightly anesthetized with isoflurane (3.5%). Normothermia was not required due to the rapid nature of the procedure. The injury apparatus consisted of a 1 m vertical metal tube and a 97 g steel weight (1.2 cm in diameter) suspended by a nylon thread. The mice were positioned on a Plexiglas box with a nicked aluminum foil top. The weight was placed just above the animal’s head, raised to a height of 1 m and then released. The impact caused the mouse to break through the aluminum foil, rotate 180 degrees, and land on a foam pad at the base of the Plexiglas box. Mild TBIs were administered twice daily, with 6 h interval, over 5 consecutive days. Sham controls underwent identical handling procedures without head trauma. Post-procedure, mice were returned to their cages for recovery. No respiratory distress or seizures were observed, and all animals survived the repetitive mTBIs. Detailed timelines for all experimental procedures are illustrated in Figure 1.

### 2.3. Tissue Preparation

For Western blotting analyses, spinal cords were rapidly extracted from the experimental animals, frozen in liquid nitrogen and stored at −80 °C until further analysis. Separate cohorts of mice were used for the histochemical assays. At the designated time points (Figure 1), these mice were intraperitoneally anesthetized with a ketamine and xylazine mixture (100 mg/kg and 10 mg/kg, respectively), followed by transcardial perfusion with ice-cold phosphate-buffered saline (PBS, 0.1 M, pH 7.4) and, subsequently, 4% paraformaldehyde (PFA (Kemika d.d., Zagreb, Croatia)) in PBS. The spinal cords were dissected, postfixed in 4% PFA overnight, dehydrated in 20% sucrose in PBS for 24–72 h at 4 °C, and then flash-frozen in tissue freezing medium (2-methyl butane (Sigma-Aldrich, St. Louis, MO, USA)) cooled with liquid nitrogen. Cervical spinal cord cryosections were cut to a thickness of 10 µm, dried for 3 h at 50 °C and stored at −80 °C until further analysis.

### 2.4. Western Blotting

Cytosolic and nuclear fractions from the isolated spinal cords were prepared for the detection of the designated proteins’ expression levels by Western blotting, following the protocol by Yadavilli et al. [46] with minor modifications. Samples were cut into smaller pieces, washed with PBS and centrifuged at 3000 rpm for 5 min at 4 °C, discarding the supernatant. The pellets were then subjected to a series of centrifugations with lysis buffers containing enzyme inhibitors. First, the pellets were treated with lysis buffer A (25 mM HEPES (Sigma-Aldrich, St. Louis, MO, USA) [pH 7.4], 50 mM KCl (Kemika d.d., Zagreb, Croatia), 2 mM EDTA (Sigma-Aldrich, St. Louis, MO, USA), 2 mM EGTA (Sigma-Aldrich, St. Louis, MO, USA), 0.1% Triton-X (Sigma-Aldrich, St. Louis, MO, USA), 1 mM DTT (Bio-Rad, Hercules, CA, USA), 25 mM NaF (Itrij, kemični inženiring, d.o.o., Kropa, Slovenia), 1 mM NaVO_3_ (Kemika d.d., Zagreb, Croatia), 1 mM PMSF (Thermo Fisher Scientific, Waltham, MA, USA), 10 µg/mL aprotinin (Sigma-Aldrich, St. Louis, MO, USA), 10 µg/mL leupeptin (Carl Roth GmbH, Karlsruhe, Germany) and 10 µg/mL NaPP (Kemika d.d., Zagreb, Croatia)), homogenized in Dounce homogenizer (Šurlan, Medulin, Croatia) and incubated on ice for 30 min. After centrifugation at 13,375 rpm for 5 min at 4 °C, the supernatant was collected as the cytosolic fraction and stored at −80 °C. The remaining pellet was resuspended in lysis buffer B (buffer A without Triton-X) and centrifuged at 3000 rpm for 5 min at 4 °C. The supernatant was discarded, and the nuclear pellets were resuspended in the nuclei extraction buffer (buffer B with 450 mM KCl and 50% glycerol (Sigma-Aldrich, St. Louis, MO, USA)) and frozen at −80 °C for 30 min. After thawing at room temperature, the sample was centrifuged at 13,375 rpm for 15 min at 4 °C, and the supernatant was collected as the nuclear fraction and stored at −80 °C.

Protein concentrations were determined by the Bradford protein assay [47]. Equal amounts of proteins per lane were loaded on a sodium dodecyl sulfate-polyacrylamide gel, separated by electrophoresis and electroblotted to nitrocellulose membranes. Blots were blocked with 5% non-fat dry milk or bovine serum albumin (BSA) blocking buffers for one hour at room temperature. Membranes were incubated with different primary antibodies (listed in Table S1) overnight at 4 °C. The next day, membranes were incubated with appropriate biotinylated secondary antibody solutions (Table S1) for 1 h at room temperature, followed by incubation with streptavidin–horseradish peroxidase conjugate incubation for half an hour. Immunoreactive bands were visualized using the SuperSignal West Pico Chemiluminescent Substrate (Thermo Fisher Scientific, Waltham, MA, USA). Signals were detected using the Kodak Image Station 440CF, and band intensities were quantified with the Kodak 1D Image Analysis Software (Eastman Kodak, Rochester, NY, USA).

### 2.5. Histological Analysis

#### 2.5.1. Cresyl Violet Staining

For the histological evaluation of fundamental neuronal structural components in the cervical segment of the spinal cord, cresyl violet (Nissl) (BioGnost d.o.o., Zagreb, Croatia) staining was utilized (Supplementary Figure S1). Initially, frozen spinal cord sections were thawed to room temperature and subsequently dried at 50 °C for 10 min. Following this, the sections were immersed overnight in a 1:1 mixture of alcohol (Etil promet d.o.o., Sveta Helena, Croatia) and chloroform (Merck & Co., Rahway, NJ, USA). Rehydration was achieved through a graded series of alcohol concentrations (100% and 95%), culminating in distilled water. The sections were then stained in a heated, acidic 0.1% cresyl violet solution for 10 min, followed by washing with distilled water, differentiation in 95% ethyl alcohol, dehydration in 100% alcohol and clearing with xylene. Finally, the sections were mounted with Entellan^®^ (Merck, Darmstadt, Germany). Microphotographs of the cresyl violet-stained sections were taken at ×400 magnification, using an Olympus BX 51 microscope equipped with an Olympus DP 70 digital camera (Olympus, Tokyo, Japan).

#### 2.5.2. Immunofluorescence/Immunohistochemistry

The spinal cord slides were thawed to room temperature and incubated at 50 °C for 10 min. Tissue permeabilization was achieved using TBS-Triton X-100 (0.025%). Subsequently, samples underwent a 2 h incubation in a blocking buffer consisting of 5% BSA/TBS-Triton X-100 (0.025%) to minimize nonspecific binding. Slides were then exposed overnight at 4 °C to primary antibodies detailed in Table S1, diluted in a solution of 1% BSA and TBS-Triton X-100 (0.025%). The following day, sections were incubated for 1 h at room temperature with the appropriate biotinylated or fluorochrome-labeled secondary antibodies, diluted 1:200 in 1% BSA/TBS-Triton X-100 (0.025%). Sections receiving biotinylated secondary antibodies underwent an additional 20 min incubation with streptavidin conjugated to the appropriate fluorochrome.

To visualize nuclei, 4′,6-diamidino-2-phenylin-dole (DAPI) (Thermo Fisher Scientific, Waltham, MA, USA) was applied at a concentration of 1 μg/mL for 5 min at room temperature. Finally, sections were embedded in Mowiol^®^ (Hoechst, Frankfurt/M., Germany), a medium conducive to fluorescent signal preservation. Double staining of spinal cord sections followed a similar protocol, employing two primary antibodies applied separately on consecutive days. For triple immunofluorescence labeling, DAPI was included alongside double staining. Immunolabeled sections were examined using light or epifluorescence microscopy (Olympus BX 51 microscope equipped with an Olympus DP 70 digital camera, Olympus, Tokyo, Japan).

Each experimental group used tissue samples from at least three animals, analyzing a minimum of three spinal cord sections per animal for immunofluorescent analysis. The extent of microgliosis and astrocytosis was quantified by measuring the percentage of Iba1- or GFAP-immunoreactive areas. Microphotographs taken at ×400 magnification were converted to 8-bit images and auto-thresholded (with 0 representing white and 255 representing black) to distinguish positive immunoreactions from the background. This allowed for the calculation of the immunoreactive area fraction. The area fractions were then averaged for each animal and each experimental group.

### 2.6. Statistical Analyses

Data for this study were collected utilizing Microsoft Excel 2016 (Microsoft Corp., Redmond, WA, USA). Densitometric analyses of protein expression levels were performed by quantifying the relative optical densities of protein bands. These values were normalized against β-actin for cytosolic samples and subsequently expressed as a percentage of the respective control group values. For the nuclear proteins, Ponceau S staining was used as a protein loading control. Statistical analyses were conducted using Statistica software version 13.0 (StatSoft, Inc., Tulsa, OK, USA). The sample size, defined as the number of animals per group for the Western blotting and immunofluorescence analyses, was determined based on previous research findings employing complementary methodologies [39,48,49]. Namely, in our previous studies, we have investigated the impact of LFPI and rmTBI on protein expression in the mouse cortex. Utilizing the mean values obtained from these experiments across various parameters, we calculated the minimum sample size needed per group to ensure robust statistical power. Statistical comparisons between two independent groups were conducted using Student’s unpaired *t*-test. For comparisons involving multiple groups, one-way analysis of variance (ANOVA) was employed, followed by Duncan’s post-hoc multiple comparison test. The results are expressed as mean ± standard deviation (SD), and a *p*-value of less than 0.05 was deemed statistically significant. To mitigate inter-session variability in Western blotting analyses, densitometric results were adjusted using the Factor Correction program version 16.1 [50].

## 3. Results

### 3.1. Repetitive Mild, but Not Single Moderate, Traumatic Brain Injury Triggers Acute Formation of TDP-43 Phosphorylated Form

As the results of clinical and experimental studies regarding the development of TDP-43 pathology after single and repetitive brain trauma are not in accordance [15,27,38,41], here we wanted to determine pathophysiological changes of TDP-43 in the cervical part of the spinal cords of the two experimental TBI protocols. Concerning that, we used the LFPI model to represent the single moderate TBI, as it is the most used model of a single brain trauma with high reproducibility [51,52]. Kane’s rmTBI model [45] was chosen for delivering repetitive head injuries, as it is the model that allows the delivery of an impact to an unrestrained mouse that can rotate freely, as this is an essential characteristic of concussive injury in humans.

**Figure 2:**
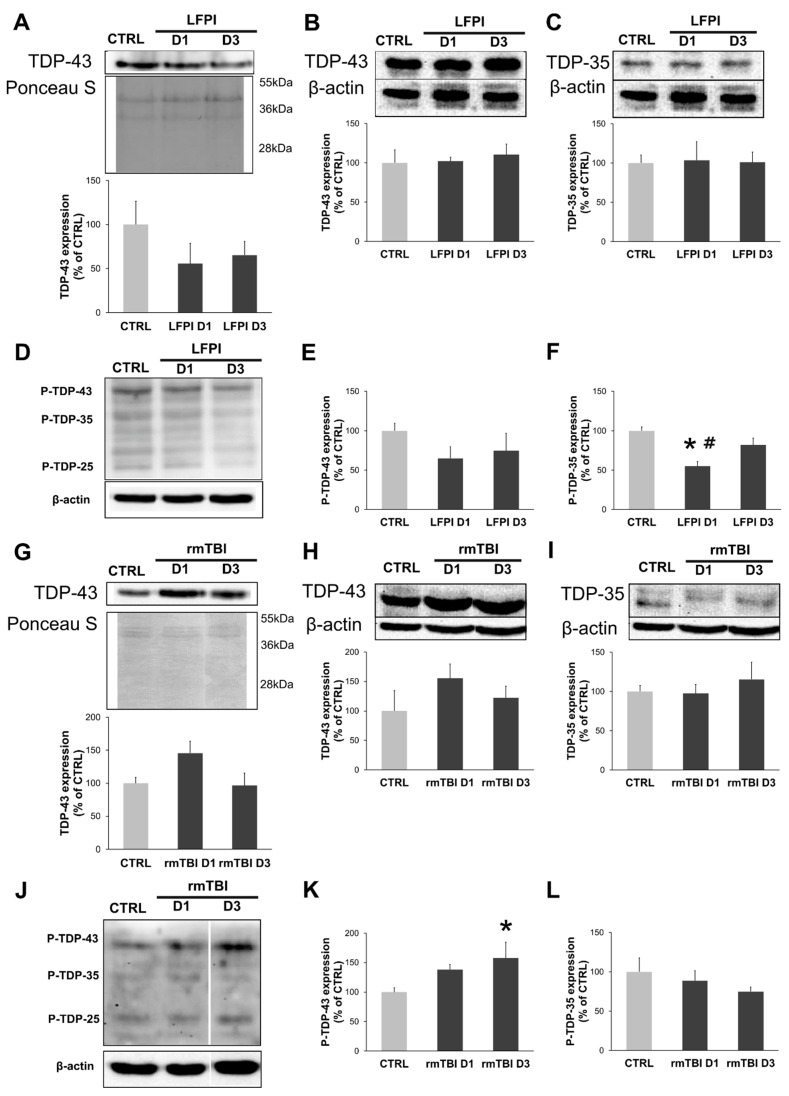
TAR DNA-binding protein 43 (TDP-43) subcellular localization, cleavage and the production of phosphorylated TDP-43 species in the cervical part of the mice spinal cord 1 and 3 days following single moderate lateral fluid percussion brain injury (LFPI) and repetitive mild traumatic brain injury (rmTBI). Representative blots and corresponding densitometric analyses of the nuclear (**A**,**G**) and cytoplasmic (**B**,**H**) TDP-43 expression levels in the mice sacrificed 1 and 3 days after LFPI or last rmTBI and the animals in the corresponding control groups (CTRL). Additionally, expression of cleaved TDP-35 was performed in cervical spinal cord samples of the animals subjected to LFPI (**C**) or rmTBI (**I**) procedures. Cytoplasmic expression of phosphorylated TDP-43 species was analyzed 1 and 3 days after LFPI or last rmTBI procedure. Representative blots and corresponding densitometric analyses of phosphorylated TDP-43 (P-TDP-43), TDP-35 (P-TDP-35) and β-actin are shown for animals exposed to LFPI (**D**–**F**) or rmTBI (**J**–**L**). In all densitometric analyses, the values are corrected for the corresponding β-actin or Ponceau S contents and expressed as % of the corresponding control groups. Error bars represent ± SD (*n* = 4–6 mice per group). * *p* < 0.05; significantly different from the related control group. # *p* < 0.05; significantly different from the LFPI D3 group.

Most experimental studies following single [23,25,41,53,54,55] and repetitive [25,40,48,53] brain injuries are focused on the acute brain changes in TDP-43 expression patterns. Here, we aimed to determine the nuclear and cytoplasmic expression patterns of TDP-43 in the cervical part of the spinal cord, in acute time points of 1 and 3 days, after single moderate and repetitive mild TBI, as shown in Figure 2.

In the cervical part of the spinal cord of the LFPI-injured animals, nuclear (Figure 2A) and cytoplasmic (Figure 2B) TDP-43 expressions showed no difference from the protein levels detected in the corresponding control animals. Also, levels of the cleaved cytoplasmic TDP-35 fragments (Figure 2C) remained unchanged when compared with corresponding control mice. In the mice subjected to repetitive mild brain injuries, protein levels of the nuclear (Figure 2G) and cytoplasmic (Figure 2H) TDP-43 did not differ from their control group. Changes in the levels of cytoplasmic TDP-35 fragments were not detected (Figure 2I). The results show that neither LFPI nor rmTBI induced acute cytoplasmic mislocalization and fragmentation.

Next, we wanted to determine if a single moderate or repetitive mild TBI could induce acute post-translational modifications of cytoplasmic TDP-43 in comparison to their corresponding brain trauma controls, as shown in Figure 2D,J. The Western blot analyses, performed with specific anti-phospho(409/410)-TDP-43 antibody, detected a significant decrease in phosphorylated TDP-35 fragment (P-TDP-35) expression on day 1 after single brain trauma (Figure 2F) [F(2,7) = 8.933; *p* = 0.012] compared to the animals of the control group (*p* = 0.004) and animals sacrificed 3 days after moderate LFPI (*p* = 0.034). Single brain trauma did not affect protein levels of phosphorylated TDP-43 (P-TDP-43) (Figure 2E) acutely. In contrast to that, repetitive mild TBIs increased P-TDP-43 (Figure 2K) protein levels on day 3 [F(2,9) = 3.016; *p* = 0.046] in comparison to the related control group. Densitometric analyses did not detect acute changes in the P-TDP-35 expression levels (Figure 2L) after rmTBI.

These results suggest that only repetitive mild, but not single moderate, TBI acutely induces the increase in the formation of abnormally phosphorylated TDP-43, in the cervical part of the spinal cord. TDP-43 proteinopathy with cytoplasmic aggregates containing P-TDP-43 species extending to the spinal cord has been observed as a common pathological finding in athletes with a concussion history [28], which is supported by the here obtained results.

### 3.2. Repetitive Mild, but Not Single Moderate, Traumatic Brain Injury Induces Prominent Subacute TDP-43 Accumulation and Pathological Post-Translational Modifications

Encouraged by the results at acute time points, we wanted to investigate whether changes in the cervical part of the spinal cord, regarding rmTBI, persist at later time periods, such as 14 days after the last mild head trauma. Also, we wanted to examine if the so-called propagation of TDP-43 proteinopathy across the neural networks to the spinal cord, as hypothesized in ALS [56], requires a longer period of time after a single moderate brain trauma, as such changes were previously detected and published by our group [39] in the region of the hippocampus.

**Figure 3:**
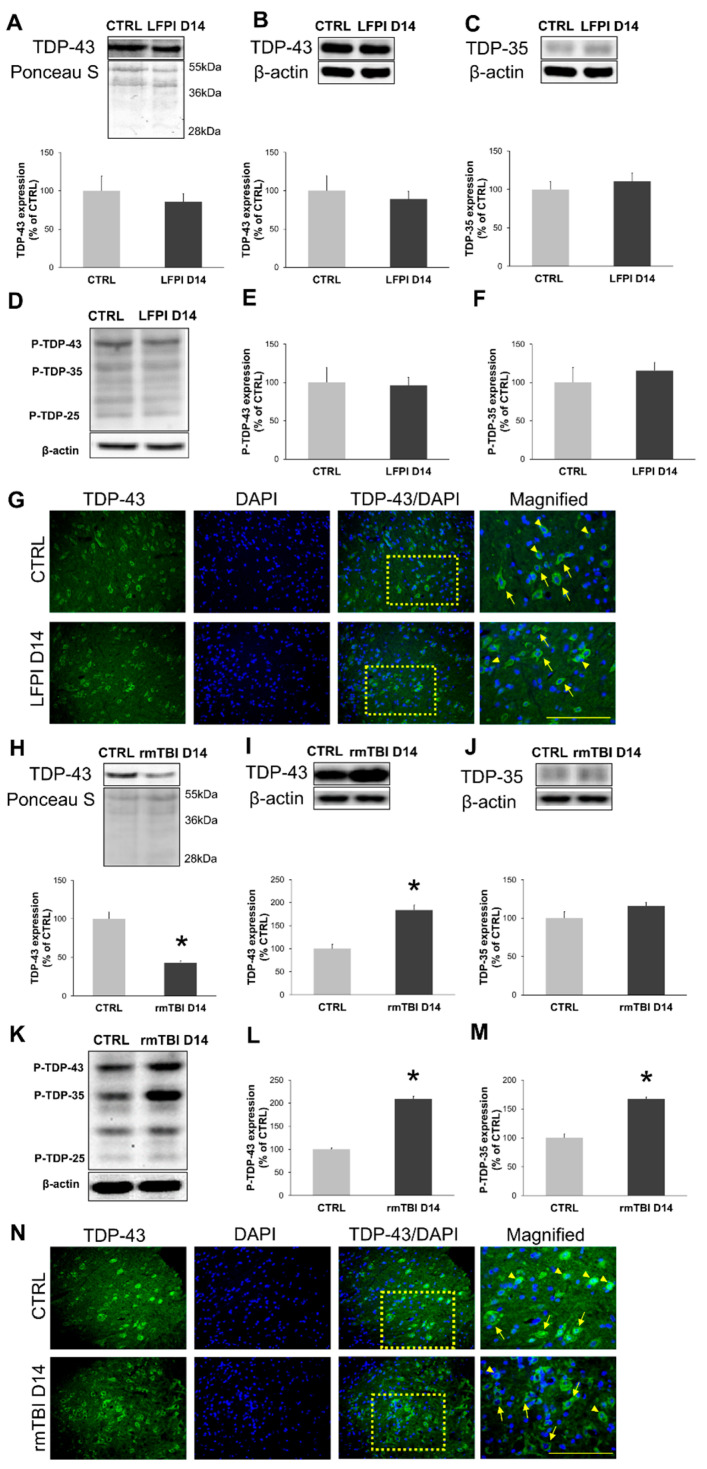
TAR DNA-binding protein 43 (TDP-43) subcellular localization, cleavage and the production of phosphorylated TDP-43 species in the cervical part of the mice spinal cord 14 days following single moderate lateral fluid percussion brain injury (LFPI) and repetitive mild traumatic brain injury (rmTBI). Representative blots and corresponding densitometric analyses of the nuclear (**A**,**H**) and cytoplasmic (**B**,**I**) TDP-43 expression levels in the mice sacrificed 14 days after LFPI or last rmTBI and the animals in the corresponding control groups (CTRL). Additionally, expression of cleaved TDP-35 was performed in cervical spinal cord samples of the animals subjected to LFPI (**C**) or rmTBI (**J**) procedures. Cytoplasmic expression of phosphorylated TDP-43 species was also analyzed 14 days after LFPI or last rmTBI procedure. Representative blots and corresponding densitometric analyses of phosphorylated TDP-43 (P-TDP-43), TDP-35 (P-TDP-35) and β-actin are shown for animals exposed to LFPI (**D**–**F**) or rmTBI (**K**–**M**). In all densitometric analyses, the values are corrected for the corresponding β-actin or Ponceau S contents and expressed as % of the corresponding control groups. Error bars represent ± SD (*n* = 4–8 mice per group). * *p* < 0.05; significantly different from the related control group. Representative microphotographs of the cervical spinal cord sections immunostained with antibody against the TDP-43 (green) and counterstained with nuclear dye (DAPI) (blue) in the animals sacrificed 14 days after LFPI (**G**) or last rmTBI (**N**) and the animals of the corresponding control group (CTRL). Scale lines: 100 µm.

Significant changes in the TDP-43 subcellular localization were not detected subacutely in the animals with LFPI when compared to their corresponding control group, as shown in the graphs of nuclear (Figure 3A) and cytoplasmic TDP-43 protein levels (Figure 3B). Immunofluorescent analysis confirmed a similar trend in the TDP-43 shift between nuclear and cytoplasmic cellular compartments between the animals subjected to LFPI and the corresponding control group (Figure 3G). Single LFPI did not induce subacute post-translational modifications of cytoplasmic TDP-43, such as fragmentation and phosphorylation (Figure 3D), as Western blot analyses did not show changes in the protein levels of cytoplasmic TDP-35 (Figure 3C), P-TDP-43 (Figure 3E) and P-TDP-35 (Figure 3F).

Contrary to that, in the animals with rmTBI, the Western blot analyses showed a significant increase in the protein expression of cytoplasmic TDP-43 (Figure 3I) (*p* = 0.014) alongside with significant decrease in nuclear TDP-43 protein levels (*p* < 0.001) (Figure 3H) 14 days after the last mild TBI, indicating cytoplasmic translocation. In addition to that, increased cytoplasmic and decreased nuclear TDP-43 levels were confirmed with immunofluorescent analysis (Figure 3N), and were susceptible to phosphorylation but not fragmentation (Figure 3J), as seen in the representative immunoblots (Figure 3K). Levels of the P-TDP-43 (Figure 3L) (*p* < 0.001) and P-TDP-35 (Figure 3M) (*p* < 0.001) 14 days after the last rmTBI were significantly higher in comparison to the related control group.

These results suggest that repetitive mild head injuries induce prominent cytoplasmic TDP-43 translocation and formation of toxic TDP-43 phosphorylated species 14 days after the last brain trauma. In the same spinal cord region, single moderate brain trauma did not promote the development of TDP-43 pathology, even at this subacute period.

At this specified subacute time point, Western blot and immunohistological analyses did not reveal changes in the amyloid precursor protein (APP) and phosphorylated TAU protein expression after single moderate or repetitive mild experimental TBI protocols, as shown in Supplementary Figure S2.

### 3.3. Choline Acetyltransferase Expression in the Cervical Part of the Spinal Cord Is Increased After Repetitive Mild, but Unchanged After Single Moderate, TBI

Acetylcholine dynamics are considered an important marker of TBI progression [57] and neuronal health [58]. Here, we wanted to examine ChAT protein expression in the cervical part of the mice spinal cord in two experimental models of head trauma.

**Figure 4:**
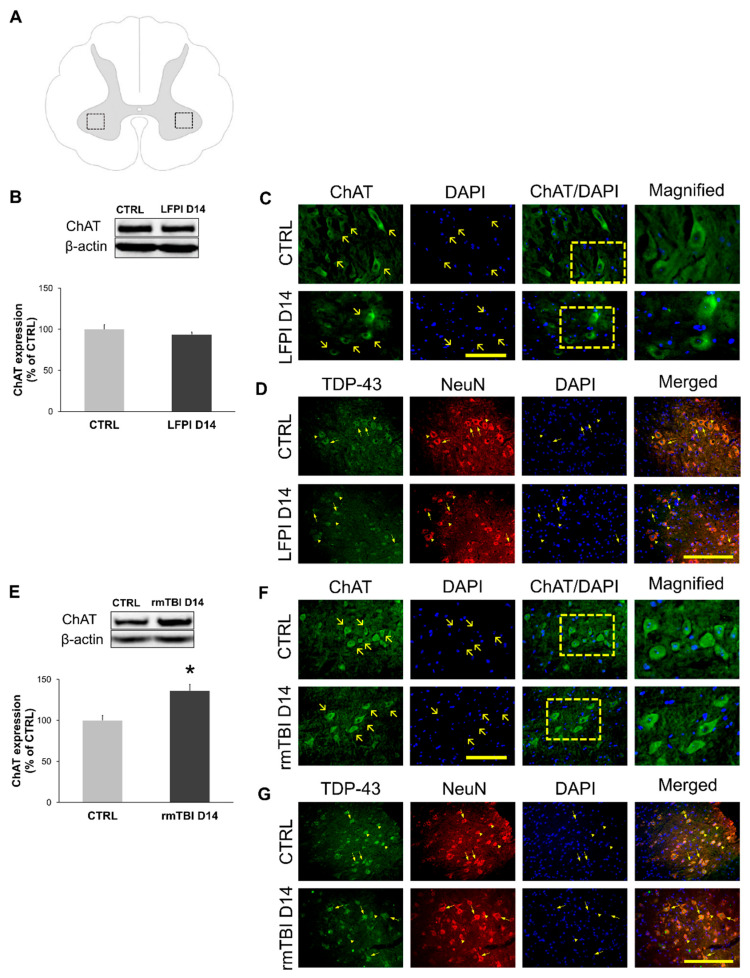
Choline acetyltransferase (ChAT) expression and cellular localization of TAR DNA-binding protein 43 (TDP-43) in the neurons of the cervical part of the mice spinal cord 14 days following single moderate lateral fluid percussion brain injury (LFPI) and repetitive mild traumatic brain injury (rmTBI). Schematic representation of the spinal cord coronal section (**A**) illustrating regions of interest. Representative blots and corresponding densitometric analyses of the ChAT expression levels in the mice sacrificed 14 days after LFPI (**B**) or last rmTBI (**E**) and the animals in the corresponding control groups (CTRL). In all densitometric analyses, the values are corrected for the corresponding β-actin contents, used as a loading control and expressed as % of the corresponding control groups. Error bars represent ± SD (*n* = 4 mice per group). * *p* < 0.05; significantly different from the related control group. Representative microphotographs of the cervical spinal cord sections immunostained with antibody against the ChAT (green) and counterstained with nuclear dye (DAPI) (blue) in the animals sacrificed 14 days after LFPI (**C**) or last rmTBI (**F**) and the animals of the corresponding control group (CTRL). Scale lines: 100 µm. Representative microphotographs of the spinal cord sections immunostained with antibodies against the TDP-43 (green), a marker of neurons (NeuN) (red) and nuclear dye DAPI (blue) in the animals sacrificed 14 days after LFPI (**D**) or last rmTBI (**G**) and their corresponding controls. Arrowheads indicate neurons with nuclear and arrows represent neurons with predominantly cytoplasmic TDP-43 staining. Scale lines: 100 µm.

Immunofluorescent detection of ChAT protein in the spinal cord motoneurons innervating the limbs was performed, as shown in Figure 4A. Western blotting analysis did not reveal differences in the ChAT protein expression in the cervical part of the spinal cord from the animals of the control group and mice sacrificed 14 following the LFPI protocol (Figure 4B). ChAT immunofluorescence did not show differences between the mice subjected to a single moderate LFPI and matching control group (Figure 4C). Further, we wanted to examine the consequences of multiple head injuries on the ChAT expression in the cervical spinal cord motor neurons. By Western blot, the protein levels of ChAT expression, at 14 days after the last repetitive injury, were revealed to be significantly elevated in comparison with the protein levels in the corresponding control animals (*p* = 0.042) (Figure 4E), which was further confirmed with immunofluorescence analysis (Figure 4F).

Previous studies demonstrated a direct interaction between TDP-43 expression and motor neuron function, indicating that motor neurons are more sensitive to TDP-43 alternations than other neurons [59]. Furthermore, we wanted to determine whether TDP-43 cellular mislocalization specifically affects motor neurons of the cervical part of the spinal cord after an individual experimental TBI protocol. Immunofluorescence analysis detected notable cytoplasmic TDP-43 staining in the motor neurons, subacutely after both single moderate (Figure 4D) and repetitive mild TBI (Figure 4G).

These results suggest that at 14 days after the last repetitive mild head injury, but not a single moderate brain trauma, an increase in the presynaptic enzyme ChAT is present. As ChAT is involved in the synthesis of acetylcholine, a crucial spinal cord neurotransmitter, these results support the hypothesis of cholinergic dysregulation after brain trauma [60]. The obtained results indicate an initial post-TBI period of cholinergic level increase, as it was previously detected in human cerebrospinal fluid and in the brains of experimental animals [60,61,62]. Taken together with the results shown in Figure 3, cytoplasmic TDP-43 mislocalization after a single moderate TBI could suggest a physiological response to injury. However, mislocalization of TDP-43 after repetitive mild head injuries, alongside increased ChAT levels, suggests potential neuronal damage.

### 3.4. Repetitive Mild Head Traumas Cause a More Extensive Subacute Microglial Activation of the Cervical Part of the Spinal Cord than a Single Moderate Brain Injury

After detecting the presence of increased levels of cytoplasmic TDP-43 and pathological P-TDP-43 and P-TDP-35 forms in the cervical part of the spinal cord following repetitive mild TBI, together with increased ChAT expression subacutely, we wanted to determine the role of microglia in the aforementioned changes. It is well known that microglia, as the resident immune cells of the brain, play a key role in the neuroinflammatory response after TBI and their activity is crucial in both neuroprotection and neurotoxicity [63]. Namely, studies have previously found that an increase in the spinal cord microglial number, including its cervical region, is evident with ALS disease progression [64]. Also, a link between microglial deregulation and motor neuron impairment in ALS has been established in previous research [65].

**Figure 5:**
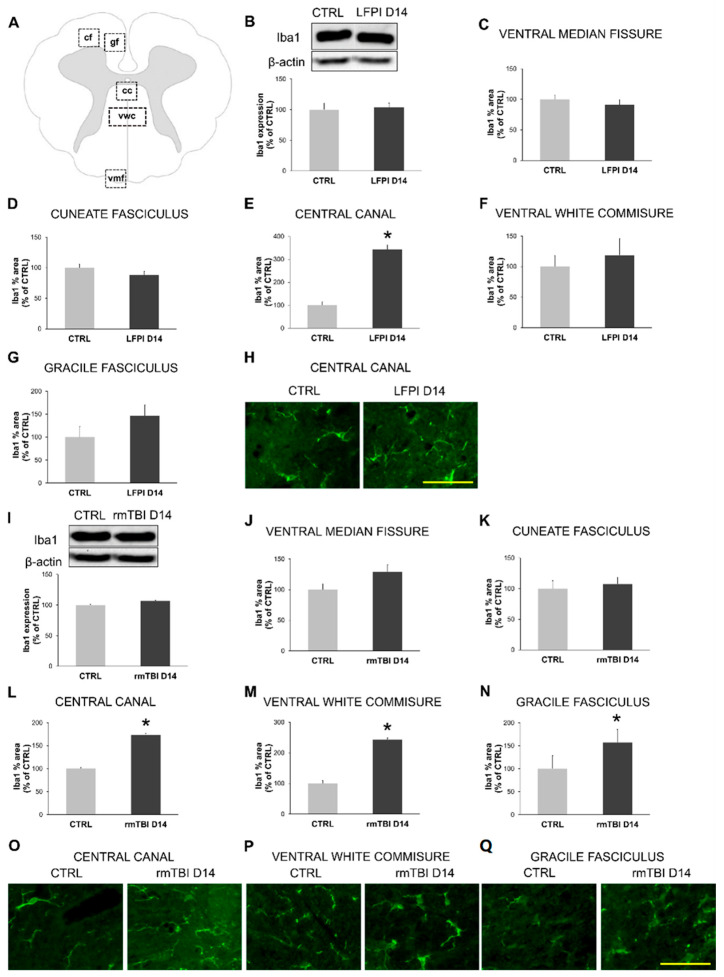
Activation of microglia in the cervical part of the mice spinal cord 14 days following single moderate lateral fluid percussion brain injury (LFPI) and repetitive mild traumatic brain injury (rmTBI). Schematic representation of the spinal cord coronal section (**A**) illustrating regions of interest as follows: ventral white commissure (vwc), ventral median fissure (vmf), central canal (cc), gracile fasciculus (gf) and cuneate fasciculus (cf). Representative blots and corresponding densitometric analyses of the total spinal cord ionized calcium-binding adapter molecule 1 (Iba1) expression levels in the mice sacrificed 14 days after LFPI (**B**) or last rmTBI (**I**) and the animals in the corresponding control groups (CTRL). In all densitometric analyses, the values are corrected for the corresponding β-actin contents that were used as loading control, and expressed as % of the corresponding control groups. Error bars represent ± SD (*n* = 4–8 mice per group). The percentage of area occupied by Iba1 in the mice sacrificed 14 days after LFPI (**C**–**G**) or last rmTBI (**J**–**N**) and the animals in the corresponding control groups (CTRL) in the aforementioned spinal cord regions of interest is shown. Values are expressed as % of the corresponding control groups, and error bars represent ± SD (*n* = 3–5 mice per group), * *p* < 0.05. Representative microphotographs of the spinal cord sections immunostained with antibody against Iba1 (green) in the animal of the corresponding control group (CTRL) and mouse sacrificed 14 days after LFPI (**H**) or last rmTBI (**O**–**Q**) in the selected regions of cervical part of the spinal cord. Scale lines: 100 µm.

Results presented in Figure 5 show microglial activation of the cervical part of the spinal cord 14 days after a single moderate or last repetitive mild TBI. Western blotting did not show differences in the protein expression of microglial marker Iba1 between mice subjected to single moderate (Figure 5B) or repetitive mild TBI (Figure 5I) and their corresponding control animals in the cervical part of the spinal cord. As it is known that there are regional differences in the distribution and molecular activity of microglial cells in the CNS [66], we decided to examine the cervical regions of the spinal cord in more detail. The percentage of the area occupied by Iba1 signal, in ventral white commissure (vwc), ventral median fissure (vmf), central canal (cc), gracile fasciculus (gf) and cuneate fasciculus (cf) was determined, as illustrated in Figure 5A.

Quantitative analysis of the Iba1 immunopositive percentage area showed a statistically significant difference in LFPI-injured animals compared with their related control group only in the region of the central canal (Figure 5E) (*p* = 0.045), while such changes were not found in the other analyzed regions (Figure 5C,D,F,G). Morphological changes that suggest microglial activation were more obvious in the animals subjected to rmTBI, as the percentage Iba1 positive area was significantly higher in the region of ventral white commissure (Figure 5M) (*p* < 0.001), central canal (Figure 5L) (*p* < 0.001) and gracile fasciculus (Figure 5N) (*p* = 0.006) when compared to the related control group. The percentage of the Iba1 positive area 14 days after the last repetitive trauma did not differ from the values of the control group animals in the regions of the ventral median fissure (Figure 5J) and cuneate fasciculus (Figure 5K).

These results indicate that multiple mild head injuries induce a more distinguished activation of microglia than a single moderate TBI in the cervical region of the spinal cord at 14 days post-injury. Further, an inflammatory response after repetitive mild TBI was confirmed with a significant increase in proinflammatory marker protein level for the cytoplasmic levels IκBα, at the same subacute time point (Supplementary Figure S3). Taken together, these results suggest that accumulation of cytosolic TDP-43, as well as pathological forms of P-TDP-43 and P-TDP-35 with an increased ChAT expression, is accompanied by the pro-inflammatory response subacutely after repetitive mild TBI′s in the cervical part of the spinal cord.

### 3.5. Significant Astrogliosis in the White and Gray Matter of the Cervical Part of the Spinal Cord Is Evident After Single Moderate and Repetitive Mild Traumatic Brain Injury

Astrocytes play a key role in the pathophysiological processes after head injury, as their beneficial and detrimental role has been confirmed in human and experimental TBI studies [67]. Astrocytes, as the CNS support cells, provide physiological spinal cord synapse functioning [68], optimal levels of calcium and potassium [69,70] and maintain the blood–brain barrier [71]. It is known that shortly after TBI, astrocytes form a glial scar at the site of injury, which is considered a chemical and physical barrier in the progression of damage [68]. Here, we hypothesize that environmental cues following brain trauma may induce an astrocytic response in the cervical part of the spinal cord.

**Figure 6:**
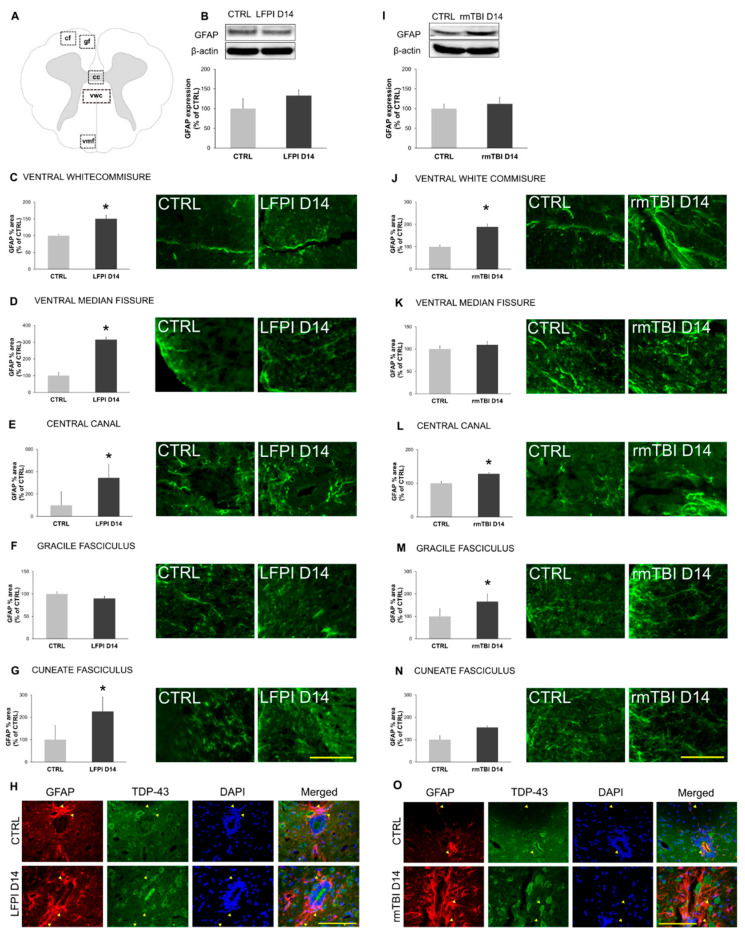
Activation of astrocytes in the cervical part of the mice spinal cord 14 days following single moderate lateral fluid percussion brain injury (LFPI) and repetitive mild traumatic brain injury (rmTBI). Schematic representation of the spinal cord coronal section (**A**) illustrating regions of interest as follows: ventral white commissure (vwc), ventral median fissure (vmf), central canal (cc), gracile fasciculus (gf) and cuneate fasciculus (cf). Representative blots and corresponding densitometric analyses of the total spinal cord glial fibrillary acidic protein (GFAP) expression levels in the mice sacrificed 14 days after LFPI (**B**) or last rmTBI (**I**) and the animals in the corresponding control groups (CTRL). In all densitometric analyses, the values are corrected for the corresponding β-actin contents, which were used as loading control, and expressed as % of the corresponding control groups. Error bars represent ± SD (*n* = 4 mice per group). The percentage of area occupied by GFAP in the mice sacrificed 14 days after LFPI (**C**–**G**) or last rmTBI (**J**–**N**) and the animals in the corresponding control groups (CTRL) in the aforementioned spinal cord regions of interest is shown. Error bars represent ± SD (*n* = 3–4 mice per group). * *p* < 0.05. Representative microphotographs of the spinal cord sections immunostained with antibody against GFAP (green) in the animal of the corresponding control group (CTRL) and mouse sacrificed 14 days after LFPI (**C**–**G**) or last rmTBI (**J**–**N**) in the selected regions of the spinal cord. Photomicrographs of the central canal spinal cord sections immunostained with antibodies against the TDP-43 (green), a marker of astrocytes (GFAP) (red) and nuclear dye DAPI (blue), in the animals sacrificed 14 days after LFPI (**H**) or last rmTBI (**O**) and their corresponding controls is shown. Arrowheads indicate astrocytes with predominantly nuclear TDP-43 staining. Astrocytes with TDP-43 cytoplasmic staining were not detected. Scale lines: 50 µm.

The astrocytic activity after single moderate and repetitive mild TBI was analyzed using the glial fibrillary acidic protein (GFAP) (Figure 6). Western blotting did not reveal statistically significant differences in the GFAP protein expression levels in the cervical part of the spinal cord 14 days after a single moderate (Figure 6B) and last mild repetitive head injury (Figure 6I). However, a detailed review of GFAP immunoreactivity at particular cervical cord regions, as shown in Figure 6A, showed regional differences in astrocytosis.

Morphological changes of GFAP positive astrocytes after single moderate LFPI were detected in the area of ventral white commissure (Figure 6C) (*p* = 0.034), ventral median fissure (Figure 6D) (*p* = 0.012), central canal (Figure 6E) (*p* < 0.001) and cuneate fasciculus (Figure 6G) (*p* = 0.023), while such changes were not detected in the area of gracile fasciculus (Figure 6F). Astroglial immunoreactivity in the animals subjected to rmTBI was significantly higher when compared with their corresponding control animals in the area of the ventral white commissure (Figure 6J) (*p* = 0.022), central canal (Figure 6L) (*p* = 0.024) and gracile fasciculus (Figure 6M) (*p* = 0.047). Quantitative analysis did not reveal astrogliosis after repetitive brain traumas in the area of the ventral median fissure (Figure 6K) and cuneate fasciculus (Figure 6N).

These results indicate that both single moderate brain injury and multiple mild head traumas induce similar extent of the astrocytic activation in the cervical part of the spinal cord at 14-days post-injury, including astrocytosis in the central canal and white matter regions. Activation of the astroglial cells after TBI was previously detected, as GFAP and S100-B can serve as a potential cerebrospinal fluid biomarker, even after single mild injury [72]. Also, studies have shown that astrocytes play an important role in ALS pathogenesis and, in the symptomatic phase of the disease, are characterized by an GFAP increase [73]. However, motor neurons have been found to be more vulnerable to TDP-43 pathology than astrocytes in ALS in vitro models [74]. Our results support these findings, as cytoplasmic expression of TDP-43 in the astrocytes was not detected in mice subjected to single moderate (Figure 6H) or repetitive mild TBI (Figure 6O), or their designated control animals.

## 4. Discussion

In the presented investigation, the effects of a single moderate and repetitive mild brain trauma on the development of pathological changes in the cervical part of mice spinal cord were studied. Research focus was on the differences between the two TBI models in terms of the induction of changes in the TDP-43 expression patterns, as well as on the neuronal, microglial and astrocytic reaction, within the first two weeks following the brain trauma procedures. We detected significant pathological changes related to TDP-43 protein in the spinal cords of animals exposed to rmTBI, which were not found in LFPI mice. Microglial activation was detected in the cervical spinal cords of animals of both TBI groups; however, it was more widespread in mice that went through repeated mTBI protocol. Contrarily, reactive astrogliosis has been detected in different regions of the cervical spinal cord samples caused by both TBI types.

For the single moderate TBI, we used the LFPI model as it is considered as a highly reproducible brain trauma model that combines both focal and diffuse aspects of this type of injury that are commonly seen in patients that have suffered falls or traffic accidents. Repetitive mild brain traumas are often seen in athletes involved in contact sports, military personnel, and victims of domestic violence, hence this type of injury was replicated in mice by employing the weight drop model, as described by Kane et al. [45] Kane’s model [45] can be considered clinically relevant because, after the induction of trauma, it allows free movement of the mouse head and body, which mimics an injury that includes a concussion.

Results reported in our study are in accordance with previous studies in which the effects of either single brain trauma or repeated head impacts were investigated in genetically ALS predisposed animals [75,76]. Namely, using the SOD1^G93A^ rat model that recapitulates human ALS, Thomsen et al. [76] demonstrated that one-time acute focal injury, caused by controlled cortical impact, does not affect disease onset or survival. Conversely, the same group showed, in the same strain of genetically modified rats, that animals with more severe injury-related deficits also presented with alterations in corticospinal motor neuron health and significantly earlier disease onset and shortened lifespan [75].

In terms of the consequences of TBI, they are believed to be the results of the secondary injury processes that involve cascading events leading to changes not only in the brain but also the rest of the CNS. As for the repeated brain traumas, it is hypothesized that this secondary injury is exacerbated by incomplete recovery between the impacts which leads to progressive damage over time.

As previously established by several studies, including our own, TBI pathophysiology involves changes in the subcellular localization and post-translational modifications of a ubiquitous TDP-43 protein [23,39,42]. Considering these findings, as well as the studies suggesting that head injuries could increase ALS and other motor neuron disease risk [28,75,77,78,79], we wanted to examine whether the different types of brain trauma pose a risk for neurodegenerative changes in the spinal cord.

In our previous study, we detected that different types of brain trauma may cause diverse pathophysiological changes in the brain, specifically related to the TDP-43 protein fate [39]. Specifically, in the hippocampus, a somewhat remote region from the directly impacted brain surface, at the 2-week post-injury time point, we detected pathological alterations only after single moderate TBI but not following rmTBI. Similarly, here we detected differences in the reaction in the cervical spinal cord between the used TBI models. Acutely, only in rmTBI, but not LFPI animals, we detected a significant increase in pathological P-TDP-43 protein expression. At 14 days following the rmTBI protocol, pronounced nucleus-to-cytoplasm shuttling of TDP-43 with an increase in the phosphorylation of both TDP-43 and TDP-35 protein forms was detected. Similar findings were not present in the analyzed samples from mice following single moderate TBI. Our findings regarding the TDP-43 in single TBI differ from those reported in the study by Bjorklund et al. [42]. Namely, using the controlled cortical impact (CCI) model of TBI, they detected TDP-43 mislocalization patterns in the cervical spinal cord, in the subacute (7 to 28 days post-TBI) as well as chronic (120 and 180 days) time points. These discrepancies might be attributed to the differences between the used single TBI models, as the CCI model is predominantly considered a focal brain injury model, with significant loss of neurons in a localized area, often times mostly in the superficial cortical layers [80]. Contrarily, in the LFPI model, neuronal damage appears more spread-out through the cortex, across different cortical areas, often extending to both ipsilateral and contralateral hemispheres [80,81,82]. Our results demonstrate a significant difference in TDP-43 mislocalization, occurring only in the rmTBI model, but not in the LFPI model, in the cervical part of the spinal cord at 14 days post-injury. Together with the findings from Bjorklund et al. [42], our results highlight the critical role of injury type in shaping the trajectory of pathological changes and their contribution to the development of subsequent neurodegenerative processes.

Pathological changes involving TDP-43, along with other RNA-binding proteins with prion-like domains, as well as modulation of stress granules’ assembly, have been linked to neurodegenerative diseases. It has been suggested that TBI might be the causative factor in the alterations in the protein clearance pathways, leading to aberrant protein aggregation as well as prion-like spreading of these toxic aggregates throughout the rest of the CNS [83]. These aggregates can spread along neuroanatomical pathways, including those that extend into the spinal cord, through synaptic connections, axonal transport or even through cerebrospinal fluid. Possible other causes of TDP-43 proteinopathy in the spinal cord post-TBI are varied and beyond the scope of this study. However, it can be hypothesized that it is the result of different processes. Namely, the traumatic events themselves, i.e., the force of impact, can cause the brain to move within the skull, potentially affecting the spinal cord at the cervical level. Post-TBI inflammatory reaction, that has been implicated already in the development of TDP-43 protein changes, may extend to the spinal cord, causing swelling and pressure. Additionally, neurochemical changes such as excitotoxicity and oxidative stress might also affect the spinal cord [84].

The cumulative pathological effect of rmTBI on the spinal cord was revealed also by measurement of ChAT expression. Namely, in the spinal cords of repetitively brain-injured animals, at 2 weeks, we detected increased expression of the enzyme. ChAT is the enzyme responsible for the acetylcholine synthesis and is considered the most specific indicator for cholinergic neurons in the central and peripheral nervous systems. In the development of most neurodegenerative diseases, ChAT expression is typically decreased, particularly in the conditions and CNS regions with significant cholinergic neurons’ loss [60,62,85]. In some instances, particularly in the early stages of diseases like ALS or in chronic pain conditions, ChAT expression might be transiently increased as a compensatory response [86,87,88]. This compensatory upregulation of ChAT in surviving cholinergic neurons is usually transient and does not prevent the progressive decline in cholinergic function. As we detected an increase in ChAT expression only in cervical spinal cords of rmTBI mice, it can be proposed that this type of traumatic event prompts neurodegenerative changes later in life.

From this study, it is evident that both types of brain trauma induced significant changes in the glial reaction in the cervical part of the spinal cord. However, there are some differences between the two TBI models. Namely, in the LFPI mice, the only region with significant microglial activity is the central canal area. Conversely, in the repetitive brain trauma model, increased microglia reaction was more widespread, involving also the regions of ventral white commissure and gracile fasciculus. The central canal-associated microglia have certain specific characteristics that differentiate it from the rest of the spinal cord microglia. These cells are situated close to the ependymal cells that line the central canal, which is filled with cerebrospinal fluid (CSF), and thus are susceptible to interactions with the ependymal cells and respond to changes in the CSF composition. As these microglial cells are implicated in the role of CSF composition monitoring, such as the alterations in cytokine levels, the presence of pathogens or other factors that indicate damage or disease, they might be present in the CSF as a consequence of the injury of other parts of the CNS [89]. Conversely, microglia in both the ventral white commissure and the gracile fasciculus are primarily involved in maintaining the integrity of axons and responding to white matter injuries. In these spinal cord regions, microglia act by releasing pro-inflammatory cytokines and other mediators that influence the local environment, potentially contributing to secondary injury or modulating repair processes, but also providing neuroprotective support to axons, aiding in remyelination or preventing further degeneration of damaged fibers. As the major component of the rmTBI model is diffuse axonal injury, the impact on the white matter tracts is higher than in the single moderate TBI. Thus, it is not surprising that we found repetitive mild brain traumas affect the microglia of the ventral white commissure and the gracile fasciculus, in addition to central canal-associated microglia. Additionally, as the microglial reaction was evident in regions with axons that primarily carry sensory information (funiculus gracilis), but also in the spinal cord region with axons that play a crucial role in the transmission of motor and pain-related signals (ventral white commissure), it can be concluded that this reaction is a generalized phenomenon and not restricted to specific sensory or motor pathways [90].

As for the astrocyte reaction, no significant difference was noted between the TBI models. In both cases, astroglia hyperactivity was apparent throughout different parts of cervical spinal cord.

In this study, a limitation was not taking into account biological variables, such as animal sex. Additionally, behavioral effects and differences were not observed, which may limit the interpretation of here stated findings.

## 5. Conclusions

In summary, this study highlights distinct pathological responses in the cervical spinal cord following different types of TBI in mice. Both single moderate TBI (LFPI model) and rmTBI (WD model) were shown to induce microglial and astrocytic activation, with rmTBI leading to more extensive microglial involvement across specific spinal cord regions. Notably, repetitive mild TBI uniquely elevated TDP-43 protein alterations and ChAT expression, indicating potential long-term neurodegenerative changes. These findings support the hypothesis that repetitive trauma exacerbates secondary injury mechanisms, possibly contributing to neurodegenerative risks. The study adds to the understanding of TBI-induced spinal cord pathologies and emphasizes the need for further research on the cumulative impacts of repeated brain injuries, particularly in populations with high exposure to such trauma.

## Data Availability

The data that support the findings of this study are available within the article and Supplemental Data or upon request from the corresponding author.

## Supplementary Materials

The following supporting information can be downloaded at: https://www.mdpi.com/article/10.3390/biomedicines13010218/s1, Table S1. List of antibodies used for the Western blotting (WB) and the immunofluorescent (IF/immunohistological (IHC) analyses; Figure S1. Histological evaluation of neuronal degeneration in the cervical part of the mice spinal cord 14 days following single moderate traumatic brain injury and last repetitive mild traumatic brain injury (rmTBI); Figure S2. Effect of a single moderate lateral fluid pressure injury (LFPI) or mild repetitive traumatic brain injury (rmTBI) on the subacute expression of amyloid precursor protein (APP) and phosphorylated tau protein (P-TAU) levels in the cervical part of the mice spinal cord; Figure S3. Expression of inflammatory markers in the cervical part of the mouse spinal cord 14 days following single moderate traumatic brain injury (LFPI) and last repetitive mild traumatic brain injury (rmTBI).
