# Supplementary material for: Repetitive Mild but Not Single Moderate Brain Trauma Is Associated with TAR DNA-Binding Protein 43 Mislocalization and Glial Activation in the Mouse Spinal Cord
Source: Biomedicines. 2025 Jan 16;13(1):218. doi: 10.3390/biomedicines13010218 (PMC11760438; doi:10.3390/biomedicines13010218)
Supplement: Supplementary file 1 [file biomedicines-13-00218-s001.zip › biomedicines-3358337-supplementary.pdf]

biomedicines

MDPI

Table S1. List of antibodies used for the western blotting (WB) and the immunofluorescent (IF/immunohistological (IHC) analyses.

Primary antibody	Structure of immunogen	Manufacturer; catalog number; host species	Concentration
anti-TDP-43	TDP-43 fusion protein	Proteintech; 10782-2-AP; rabbit polyclonal	1:2000 (WB)<br>1:200 (IF)
anti-phospho (409/410)-TDP-43	Phospho-TDP43 (Ser409/410)	Proteintech; 22309-1-AP; rabbit polyclonal	1:1000 (WB)
anti-β-actin	β-actin (C4) raised against gizzard Actin of chicken origin.	Santa Cruz Biotechnology; sc-47778; mouse monoclonal	1:2000 (WB)
anti-ChAT	Recombinant fragment corresponding to a region within amino acids 102 and 351 of Human CHAT	Thermo Fisher Scientific; PA5-29653; rabbit polyclonal	1:1000 (WB)<br>1:250 (IF)
anti-NeuN	NeuN clone (A60) Purified cell nuclei from mouse brain	Merck Millipore; MAB377; mouse monoclonal	1:100 (IF)
anti-Iba1	Synthetic peptide corresponding to Human Iba1 aa 135-147 (C terminal)	Abcam; ab5076; goat polyclonal	1:500 (WB)
anti-Iba1	Ionized calcium binding adaptor molecule 1 (IBA1) raised against synthetic peptide corresponding to C-terminus of IBA1	FUJIFILM Wako; 019-19741; rabbit polyclonal	1:500 (IF)
anti-GFAP	Recombinant full length protein corresponding to Human GFAP. Isotype 1 expressed in and purified from *E. coli*	Abcam; ab7260; rabbit polyclonal	1:10 000 (WB)
anti-GFAP	total GFAP protein (full length)	Cell Signaling Technology; 3670; mouse monoclonal	1:100 (IF)
anti- P-p65	Phospho-NF-kappaB p65 phosphorylated at Ser536	Cell Signaling Technology; 3033; rabbit monoclonal	1:500 (WB)
anti-IκBα	A synthetic peptide corresponding to human IκBα	Abcam; ab32518; rabbit monoclonal	1:5000 (WB)
anti-iNOS	Synthetic peptide corresponding to Mouse iNOS aa 1-100	Abcam; ab3523; rabbit polyclonal	1:1000 (WB)
anti-TLR2	Recombinant fragment. Immunogen information is proprietary to Abcam	Abcam; ab209217; rabbit monoclonal	1:1000 (WB)
APP	Synthetic peptide corresponding to N terminal amino acids 44-62 of Human Amyloid precursor Protein	Abcam; ab15272; rabbit polyclonal	1:1000 (WB)<br>1:100 (IHC)
P-TAU	Phospho-Tau (Thr181) (D9F4G) recognizes endogenous levels of Tau protein only when phosphorylated at Thr181	Cell Signaling Technology; 12885; mouse monoclonal	1:1000 (WB)<br>1:200 (IHC)
**Secondary antibody**		**Manufacturer; catalog number; host species**	**Dilution**
biotinylated goat anti-mouse	Gamma Immunoglobins Heavy and Light chains	Thermo Fisher Scientific; A16070; goat polyclonal	1:20 000(WB)<br>1:200 (IF)
biotinylated goat anti-rabbit	Gamma Immunoglobin	Thermo Fisher Scientific; 65-6140; goat polyclonal	1:20 000 (WB)<br>1:200 (IF)

biotinylated rabbit anti-goat	Gamma Immunoglobin	Thermo Fisher Scientific; 31732; rabbit polyclonal	1:20 000 (WB)
goat anti-rabbit Alexa Fluor 488	Gamma Immunoglobins Heavy and Light chains	Thermo Fisher Scientific; A-11034; goat polyclonal	1:200 (IF)
donkey anti-rabbit Alexa Fluor 488	Heavy and Light chains	Abcam; ab150061; rabbit polyclonal	1:200 (IF)
donkey anti-goat Alexa Fluor 594	Heavy and Light chains	Abcam; ab150061; goat polyclonal	1:200 (IF)

Abbreviations: TDP-43, TAR DNA binding protein 43; phospho(409/410)-TDP-43, phosphorylated TDP-43; ChAT, Choline Acetyltransferase; NeuN, neuronal nuclei protein; Iba-1, ionized calcium-binding adapter molecule 1; GFAP, glial fibrillary acidic protein; P-p65, phosphorylated p65 subunit of nuclear factor kappa B (P-p65); IκBα, nuclear factor of kappa light polypeptide gene enhancer in B-cells inhibitor alpha; iNOS, inducible nitric oxide synthase; TLR2, Toll-like receptor 2; APP, amyloid precursor protein; P-TAU, phosphorylated TAU protein.

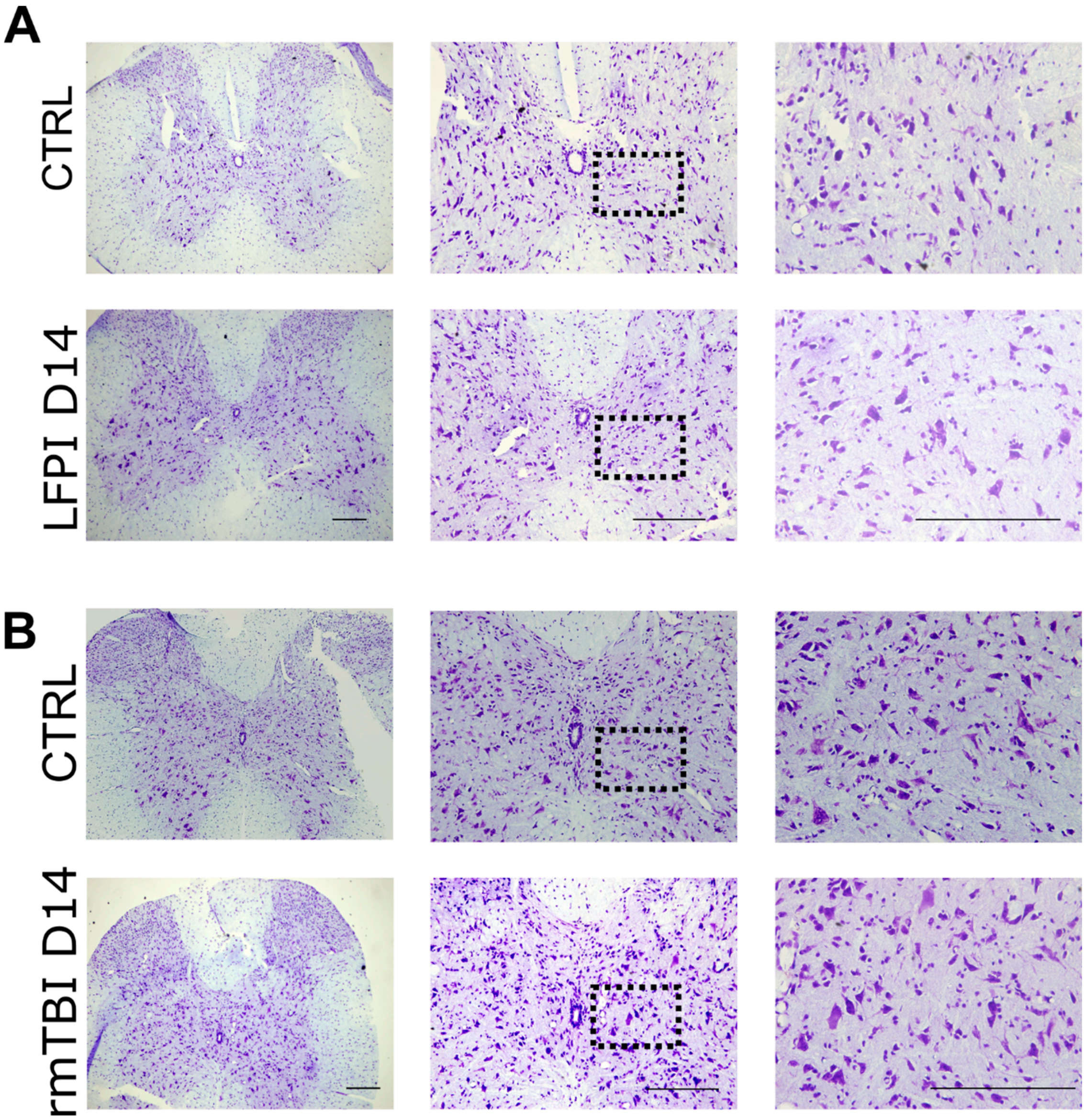

Figure S1. Histological evaluation of neuronal degeneration in the cervical part of the mice spinal cord 14 days following single moderate traumatic brain injury and last repetitive mild traumatic brain injury (rmTBI). Representative microphotographs of the cresyl violet (Nissl) stained cervical spinal cord sections of the animals sacrificed 14 days after the single moderate traumatic brain injury (LFPI D14) or last mild repetitive brain trauma (rmTBI D14) and the corresponding sham-injured mice (CTRL). Squares indicate regions of gray matter with abundant dark stained neurons. Scale bar = 200 µm at 10×, 20× and 40× magnification (from left to right). Major histological differences between the cervical spinal cords in the mice of the LFPI or rmTBI in comparison with their corresponding control groups were not observed.

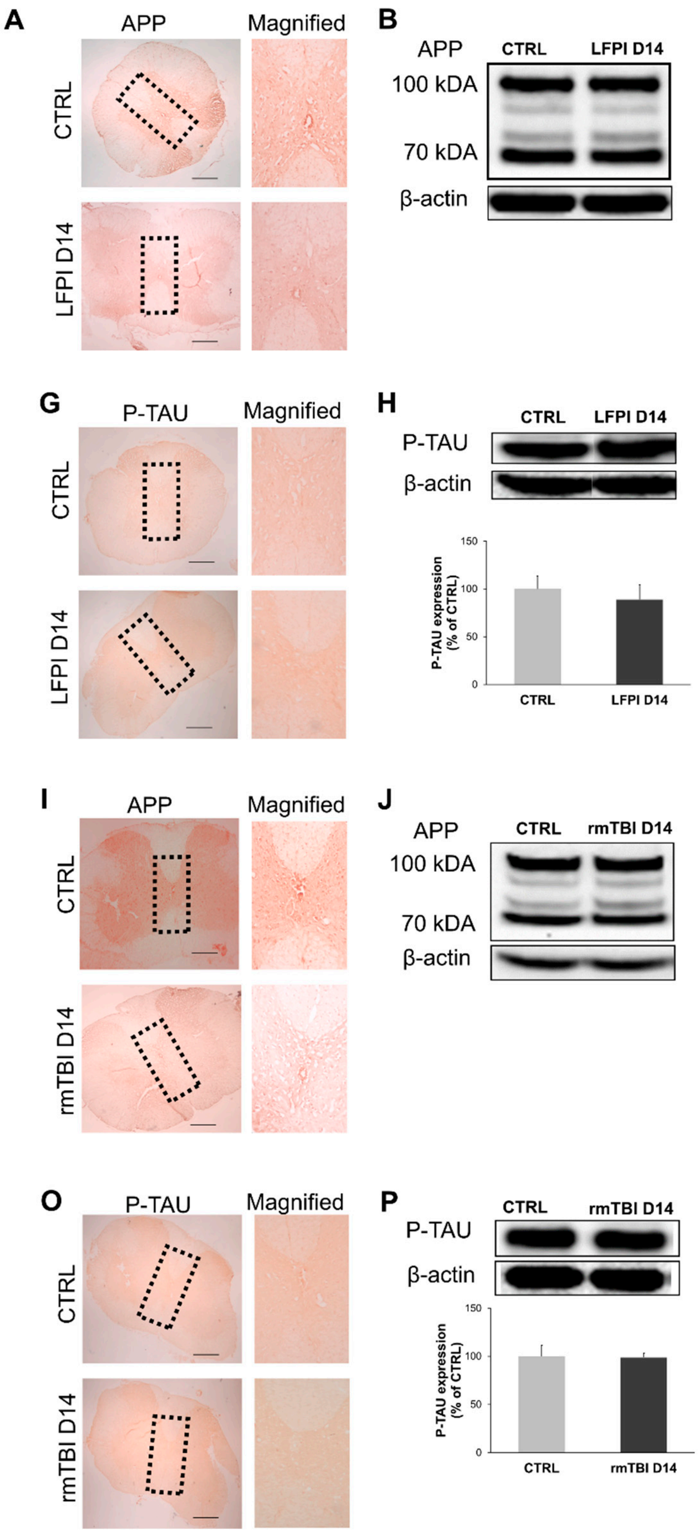

Figure S2. Effect of a single moderate lateral fluid pressure injury (LFPI) or mild repetitive traumatic brain injury (rmTBI) on the subacute expression of amyloid precursor protein (APP) and phosphorylated tau protein (P-TAU) levels in the cervical part of the mice spinal cord. Representative photomicrographs of the indicated regions immunohistologically labeled with an antibody against APP in animals of related control groups (CTRL) and mice sacrificed 14 days after LFPI (**A**) or last rmTBI (**I**) did not show any changes among tested groups. Representative blots (**B**;**J**) and corresponding densitometric analyses for APP for mice subjects to LFPI (**C**,**D**,**E**,**F**) or rmTBI (**K**,**L**,**M**,**N**) and their corresponding control groups (CTRL) confirmed immunohistological analyses. Representative

photomicrographs of immunohistologically labeled cervical spinal cord sections against P-TAU of mice subjected to LFPI (**G**) or rmTBI (**O**) and designated controls did not reveal any differences among tested groups. Representative blots and corresponding densitometric analyses for P-TAU for LFPI (**H**) and rmTBI (**P**) confirmed these findings. Scale lines: 200 μm, magnification 20×. In all the densitometric analyses, the results were corrected for the values of β-actin (cytoplasmic loading control) and expressed as % of the related control groups. Error bars represent ± SD (*n*=4 mice per group).

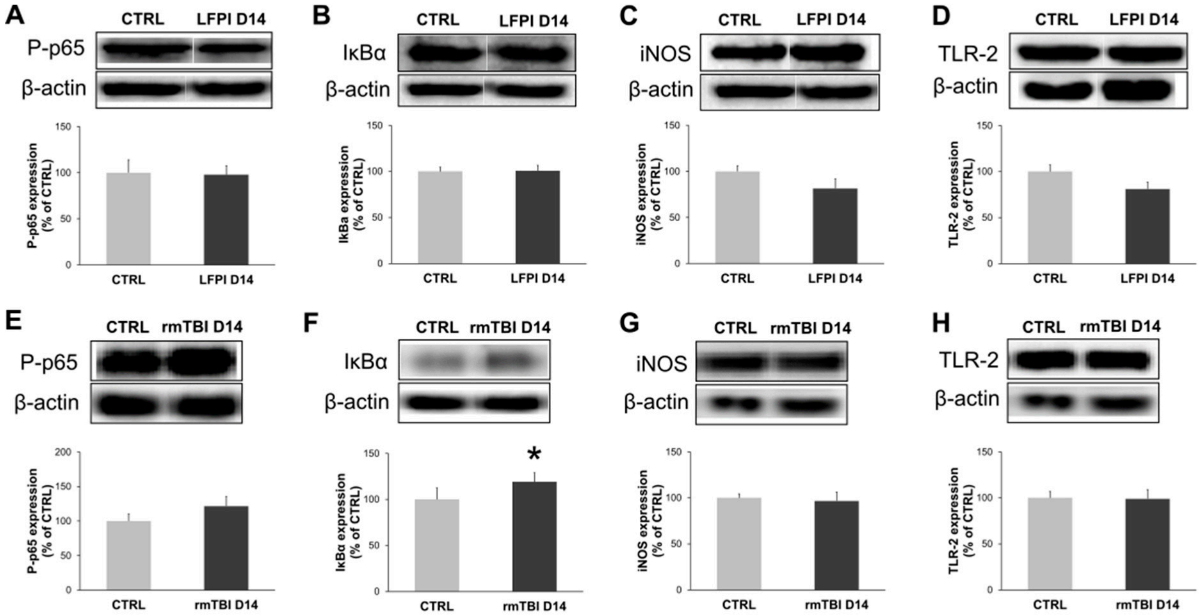

Figure S3. Expression of inflammatory markers in the cervical part of the mice spinal cord 14 days following single moderate traumatic brain injury (LFPI) and last repetitive mild traumatic brain injury (rmTBI). Representative blots and corresponding densitometric analyses for phosphorylated p65 subunit of nuclear factor kappa B (P-p65), nuclear factor of kappa alpha light polypeptide gene enhancer in B-cells inhibitor (IκBα), inducible nitric oxide synthase (iNOS), Toll-like receptor 2 (TLR2) and β-actin in the animals of related control groups (CTRL) and mice sacrificed 14 days after LFPI (**A**,**B**,**C**,**D**) or last rmTBI (**E**,**F**,**G**,**H**). Significant changes in the aforementioned inflammatory markers were not detected for the mice subjected to LFPI. In the animals subjected to rmTBI, a significant increase in the cytoplasmic IκBα levels were detected. In all the densitometric analyses, the results were corrected for the values of β-actin (cytoplasmic loading control) and expressed as % of the related control groups. Error bars represent ± SD (n=4-8 mice per group).
